# Evaluating the Suitability of Four Plant Functional Groups in Green Roofs Under Nitrogen Deposition

**DOI:** 10.3390/plants15010043

**Published:** 2025-12-23

**Authors:** Nan Yang, Hechen Li, Runze Wu, Yihan Wang, Meiyang Li, Lei Chen, Hongyuan Li, Guang Hao

**Affiliations:** 1 School of Life Science, North China University of Science and Technology, Tangshan 063210, China; yangnan079@ncst.edu.cn (N.Y.); lihechen@stu.ncst.edu.cn (H.L.); wurunze@stu.ncst.edu.cn (R.W.); wangyihan@stu.ncst.edu.cn (Y.W.); 2 Policy Research Center for Environmental and Economy, Ministry of Ecology and Environment, Beijing 100101, China; li.meiyang@prcee.org; 3 College of Life Science, Nankai University, Tianjin 300071, China; leochen@nankai.edu.cn; 4 College of Environmental Science & Engineering, Nankai University, Tianjin 300350, China; eialee@nankai.edu.cn

**Keywords:** growth performance, aesthetic value, plant functional traits, green roofs, nitrogen addition

## Abstract

The rapid urban expansion in the past few decades has resulted in a deficit of urban green space, and green roofs have become an effective way to expand urban green spaces. High nitrogen (N) deposition induced by urban development has threatened the health and sustainability of plants. The aim of this study was to evaluate the responses of plant growth performance and aesthetic value to N deposition in green roofs. Eleven species from four plant functional groups were grown under control, low N addition, and high N addition conditions to assess the effects of N addition on their growth performance, aesthetic value, soil properties, and plant functional traits. Different plant functional groups exhibited distinct traits, and their response to N addition was different. Under high N addition, the growth performance of sod-forming graminoids and tall forbs decreased by 47.0% and 23.7%, and their aesthetic value decreased by 24.4% and 16.2%, respectively. Growth performance of plant functional groups was mainly determined by plant functional traits rather than soil properties. The poor growth performance and aesthetic value of sod-forming graminoids and tall forbs challenged their widespread use under high N addition. This study highlighted the importance of selecting environmentally adaptive species from the perspective of plant functional groups, especially in the context of future high N deposition.

## 1. Introduction

Green roofs can be simply defined as engineered ecosystems constructed on the top of a building to accommodate vegetation growth in a designated substrate [1,2]. Due to urban expansion leading to a reduction in green spaces and severe environmental damage, implementing green roofs is a particularly promising approach for improving urban conditions (i.e., nature-based solutions, NbS) [1,3,4]. Indeed, green roofs provide multiple social, economic, and environmental benefits in cities, including the reduction in electricity consumption by reducing indoor temperatures [5], enhanced carbon sequestration [4], urban heat island mitigation [6], urban flash flood control [7], habitat creation for biodiversity [8], and elevated quality of life for residents [9]. The realization of these ecological functions depends on the growth performance and health status of the rooftop plants. However, habitat conditions on green roofs are usually harsh relative to comparable ground-level habitats, such as shallow soil with limited water-holding capacity and drastic fluctuations in moisture and temperature caused by high irradiance and wind exposure [10], making them fragile and susceptible to environmental changes [4]. Recognizing the impact of environmental changes on green roofs will enable urban managers to better protect and manage these engineering ecosystems.

Since the industrial revolution, anthropogenic activities have led to severe nitrogen deposition, which has caused a significant global ecological and environmental problem over the past century [11]. Nitrogen deposition is suggested to remarkably alter the availability of soil nitrogen [12,13], shift plant resource acquisition strategy, and affect plant growth and health [14,15,16]. Based on natural ecosystems, nitrogen enrichment has different effects on plant growth and health, mainly depending on the saturation level of nitrogen in the soil and the interactions with other limiting factors [12,13,17]. Compared to natural ecosystems, green roofs are replaced with shallow artificial substrates instead of soil, which typically have a lower nutrient content [5,6]. Therefore, the response of plant growth to nitrogen deposition may differ from that of natural ecosystems, but there is currently little research considering the impact of this environmental change [18].

Selecting suitable plants is the key to the sustainability and optimal performance of green roofs [3]. Thus, it should be designed accurately, considering not only the environmental conditions but also the potential benefits. Succulent species (such as *Sedum* spp.) are well-suited to survive under harsh conditions and are the most commonly used green roof plants worldwide [5,6]. However, these species typically have lower water consumption, which is a positive characteristic in terms of sustainability, but also limits the effectiveness of reducing runoff [3]. Recent research suggests that incorporating forbs and graminoids into green roofs may offer advantages over succulents, attributed to their superior stomatal conductance and biomass accumulation [4,19]. Usually, plants from the same functional group have similar performance [20,21]. Thus, understanding the response of different functional groups to environmental changes can help planners choose suitable plant planting plans more simply and efficiently.

Plants of different functional groups have specific functional characteristics [20,22], and they respond differently to environmental changes [16]. Specifically, plant functional groups may vary in their sensitivity to nutrient availability changes; thus, N enrichment would impact plant nutrition and growth in a functional group-specific manner [13,14,16,21]. For instance, succulents exhibit slow growth and high-stress resistance to various abiotic stresses by regulating osmotic stress [3,23,24]; thus, they may be less affected by nitrogen enrichment. While graminoid species (such as Poaceae and Cyperaceae) are sensitive to changes in nutrients, their growth may be enhanced under nitrogen enrichment [25]. However, it is unclear whether there are distinct response patterns among different plant functional groups to nitrogen enrichment in green roofs. Recent studies have shown that plant functional traits reflect the ability of plants to utilize resources and adapt to environmental changes [4,20], often used to explain the underlying links between environmental change and various ecological processes in plants [26,27]. Plant functional groups have similar trait combinations or assemblages [21]. Thus, examining the responses to environmental changes in traits would provide theoretical guidance for different functional groups to adapt to environments.

The green roof industry in China has developed rapidly, but few studies about suitable plant functional groups and their responses to global change in green roofs have been reported [1], especially in North China. This study aims to evaluate the suitability of four different plant functional groups under different nitrogen addition treatments and analyze which biotic and abiotic factors affect plant growth and aesthetic value. Specifically, we proposed two hypotheses: (1) N addition has different effects on the growth performance and aesthetic value of four plant functional groups, with fast-growing groups being significantly affected, while slow-growing groups (such as succulents) being less affected, and (2) the growth performance and aesthetic value of four plant functional groups was mainly determined by the changes in their traits and soil physicochemical properties.

## 2. Results

### 2.1. Effects of N Addition on Growth and Aesthetic Value of Four Functional Groups

**Figure 1:**
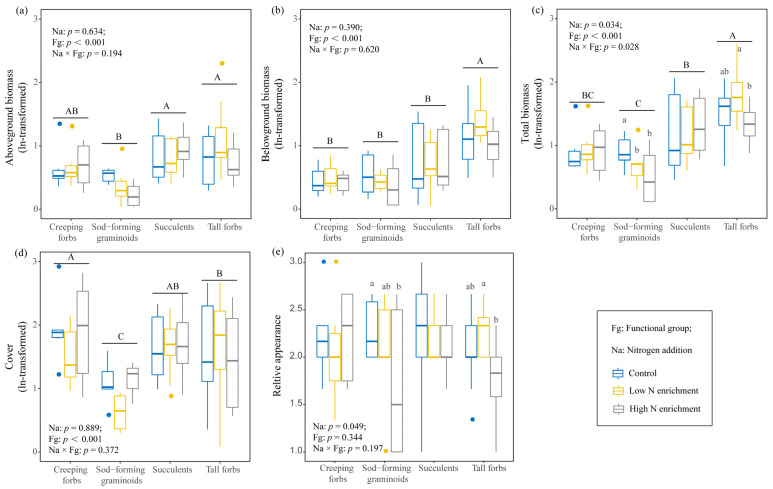
Effects of N addition on aboveground biomass (**a**), belowground biomass (**b**), total biomass (**c**), cover (**d**), and relative appearance (**e**) of different plant functional groups. The same lowercase letters represent a non-significant difference (*p* > 0.05) among different N addition treatments, and the uppercase letters represent a non-significant difference (*p* > 0.05) among different plant functional groups.

N addition affected the total biomass and relative appearance of different functional groups (Figure 1). In particular, high N addition decreased total biomass of sod-forming graminoids and tall forbs by 47.0% and 23.7%, and relative appearance by 24.4% and 16.2% (Figure 1c,e). Significant differences were observed in biomass and cover among different functional groups, in which tall forbs have higher biomass and cover, while sod-forming graminoids have lower biomass and cover (Figure 1a–d).

### 2.2. Effects of N Addition on Soil Properties of Four Functional Groups

**Figure 2:**
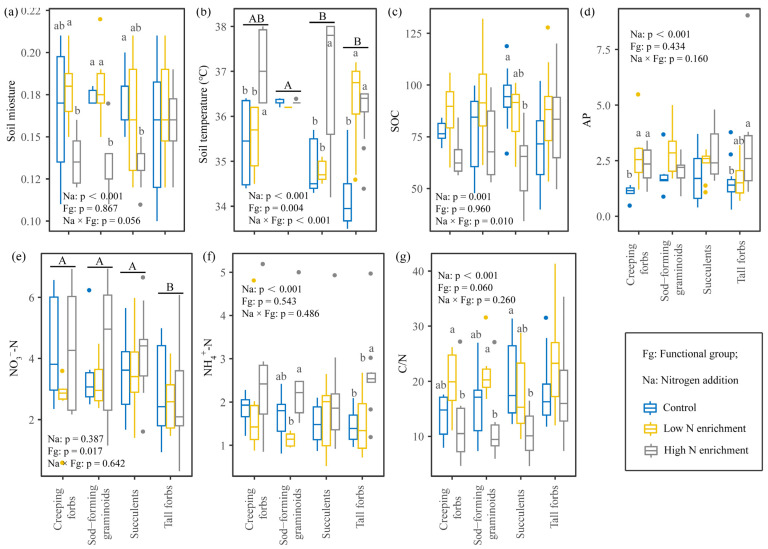
Effects of N addition on soil physicochemical properties of different functional groups. (**a**) Soil moisture; (**b**) soil temperature; (**c**) SOC, soil organic carbon; (**d**) AP, available phosphorus; (**e**) NO_3_^−^-N, nitrate nitrogen; (**f**) NH_4_^+^-N, ammonium nitrogen; and (**g**) C/N, the ratio of soil carbon to nitrogen. The same lowercase letters indicate non-significant differences (*p* > 0.05) among different N addition treatments; and the same uppercase letters indicate non-significant (*p* > 0.05) differences among different plant functional groups.

N addition had different effects on soil properties of different plant functional groups (Figure 2). Soil moisture of creeping forbs, sod-forming graminoids, and succulents was lower in high N addition, while soil temperature of creeping forbs, succulents, and tall forbs was higher in high N addition (Figure 2a,b). N addition decreased organic carbon of succulents and C/N of creeping forbs, sod-forming graminoids, and succulents (Figure 2c,g), but increased AP of creeping forbs and tall forbs and NH_4_^+^-N of sod-forming graminoids and tall forbs (Figure 2d,f).

### 2.3. Effects of N Addition on Functional Traits of Four Functional Groups

**Figure 3:**
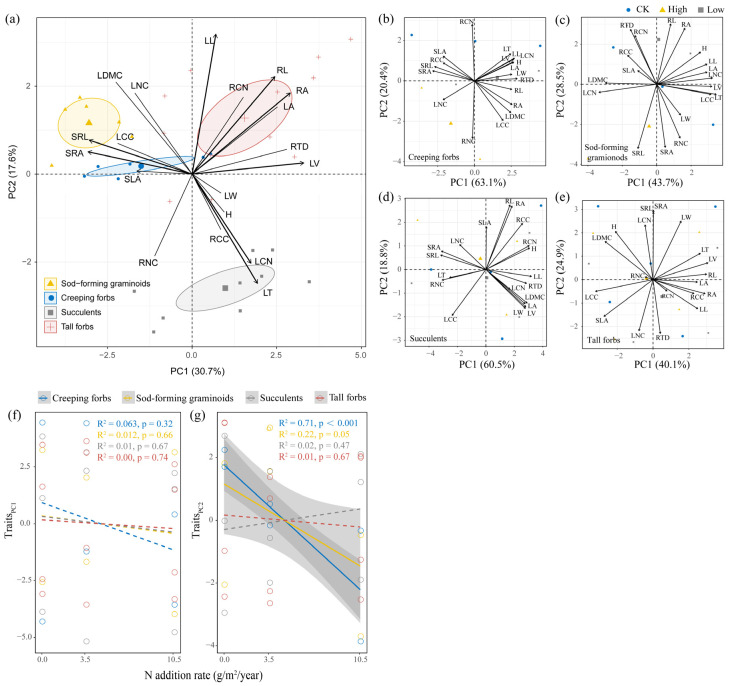
Principal component analysis (PCA) for whole and individual plant functional groups (**a**–**e**) and the relationships between functional traits and N addition (**f**,**g**). Trait acronyms: LA, leaf area; LL, leaf length; LW, leaf width; LT, leaf thickness; LV, leaf volume; SLA, specific leaf area; LDMC, leaf dry matter content; LNC, leaf nitrogen content; LCC, leaf carbon content; LCN, leaf C/N ratio; H, plant height; RL, root length; RA, root area; RTD, root tissue density; SRL, specific root length; SRA, specific root area; RNC, root nitrogen content; RCC, root carbon content; RCN, root C/N ratio. Solid and dashed lines indicate significant and nonsignificant fits in (**f**,**g**), respectively.

Four plant functional groups exhibit distinct traits (Figure 3a). Creeping forbs have higher specific leaf area (SLA) and lower height. Sod-forming graminoids have higher specific root length (SRL) and specific root area (SRA). Succulents have thicker leaves and a higher leaf C/N ratio (LCN). Tall forbs have higher root length (RL) and root area (RA). Results of PCA analysis on four plant functional group traits revealed that the first two principal components accounted for 65.0–83.5% of the variability in traits (Figure 3b–e). For creeping forbs, PC1 and PC2 traits were characterized by high root tissue density (RTD), root C/N ratio (RCN), and low SRA and root nitrogen content (RNC), suggesting a conservative strategy for nutrient absorption and nitrogen utilization efficiency (Figure 3b). For sod-forming graminoids, the PC1 axis opposed leaf nitrogen content (LNC) and LCN, reflecting trade-offs between photosynthetic rate and stress resistance. The PC2 axis, which opposed RTD and SRL, was interpreted as representing root nutrient absorption trade-offs (Figure 3c). For succulents, the PC1 axis opposed RTD and SRL, reflecting root nutrient absorption trade-offs. The PC2 axis, which opposed RL and leaf carbon content (LCC), was interpreted as representing trade-offs in carbon partitioning and water absorption (Figure 3d). For tall forbs, the PC1 axis, which opposed RL and LCC, reflected carbon partitioning and water absorption trade-offs. The PC2 axis, which opposed RTD and SRA, was interpreted as representing trade-offs in root nutrient absorption (Figure 3e).

N addition did not affect PC1 traits of the four functional groups (Figure 3f), but significantly decreased PC2 traits of creeping forbs and sod-forming graminoids (Figure 3g).

### 2.4. Relationships Between Growth Performance, Aesthetic Value, and Predictor Variables of Different Functional Groups

**Figure 4:**
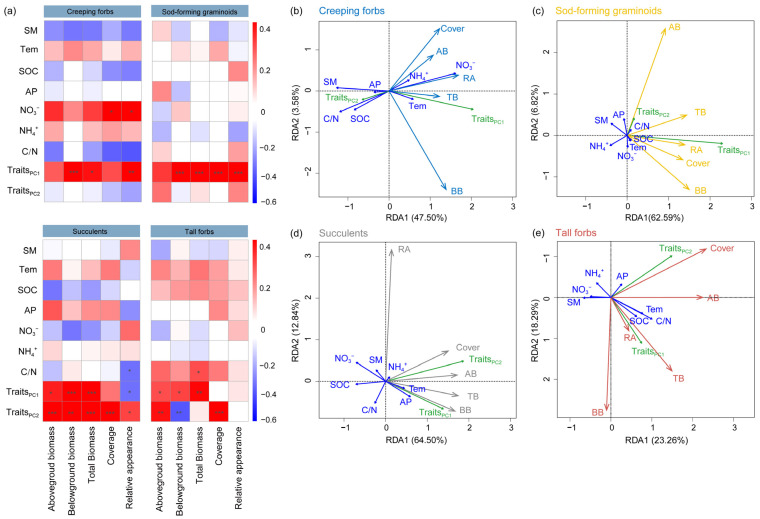
Correlation analyses (**a**) and redundancy analysis (**b**–**e**) for soil properties, functional traits, and aboveground, belowground, total biomass, cover, and aesthetic value of creeping forbs, sod-forming graminoids, succulents, and tall forbs. In (**a**), red indicates positive correlations, while blue signifies negative correlations. Significance level of correlations was represented by * (*p*  <  0.05), ** (*p*  <  0.01), and *** (*p*  <  0.001). Abbreviation: SM, soil moisture; Tem, soil temperature; SOC, soil organic carbon; AP, available phosphorus; NO_3_^−^, nitrate nitrogen; NH_4_^+^, ammonium nitrogen; C/N, the ratio of soil carbon to nitrogen; AB, aboveground biomass; BB, belowground biomass; TB, total biomass.

For creeping forbs, belowground and total biomass were positively correlated with PC1 traits. Cover was positively correlated with NO_3_^−^-N, but negatively correlated with C/N. Aesthetic value positively correlated with NO_3_^−^-N and PC1 traits (Figure 4a,b). For sod-forming graminoids, belowground biomass, total biomass, coverage, and aesthetic value were positively correlated with PC1 traits (Figure 4a,c). For succulents, aboveground biomass positively correlated with PC1 and PC2 traits. Belowground and total biomass were positively correlated with PC1 and PC2 traits, and coverage positively correlated with PC2 traits. Aesthetic value was negatively correlated with C/N and PC1 traits, but positively correlated with PC2 traits (Figure 4a,d). For tall forbs, aboveground biomass was positively correlated with PC1 and PC2 traits. Belowground biomass was positively correlated with PC1 traits, but negatively correlated with PC2 traits. Total biomass was positively correlated with C/N and PC1 traits, and coverage positively correlated with PC2 traits (Figure 4a,e). Relationships between individual functional traits and growth performance and aesthetic values were similar to those of multiple traits (i.e., PC traits, Supplementary Materials S2, Figure S2).

## 3. Discussion

Our study evaluated the suitability of four plant functional groups under the background of nitrogen deposition in experimental green roof modules in North China. Our findings indicated that N addition had different effects on four plant functional groups. Importantly, high N addition decreased the plant growth performance and aesthetic value of sod-forming graminoids and tall forbs. Soil properties had a relatively minor impact on growth performance and aesthetic value, while plant functional traits can well explain the growth performance of different plant functional groups. Specifically, RA, RL, and RTD improved plant growth performance of creeping forbs; LA, LV, and LT improved plant growth performance of sod-forming graminoids; RTD, RCN, RL, and RA improved plant growth performance of succulents; and RL and RA improved plant growth performance of tall forbs (Figure 4 and Figure S2).

N addition significantly affected the plant growth of sod-forming graminoids and tall forbs rather than other functional groups, which indicated that the response patterns of different functional groups are inconsistent, supporting our Hypothesis 1. Biomass has been proven to be a comprehensive indicator to reflect growth status and adaptive capacity of plants to changing environmental conditions [13,14,17]. High N addition decreased the total biomass of sod-forming graminoids and tall forbs in green roofs (Figure 1). Compared with other functional groups, graminoids have higher specific root length, specific root area, and leaf nitrogen content (Figure 3a), which represents a resource use strategy with nutrient uptake and assimilation [27]. In our study, we found that high leaf nitrogen content (PC1 traits) promoted the growth of graminoids (Figure 4). Generally, graminoids prefer nitrate nitrogen, with high resource utilization rates and fast growth rates, and their growth is enhanced under nitrogen enrichment [17,25]. However, the growth of graminoids was inhibited in green roofs under high N addition. This might be due to the increased water demand of graminoids. We found that high N addition promoted root nutrient uptake of graminoids (Figure 3g, PC2 traits: higher SRL, SRA, and RNC). Resource-acquiring plants require higher water to maintain their high growth rates [8,28]. Considering frequent droughts in the rooftop, this shift would reduce plant fitness and hinder their growth [13,28].

Tall forbs did not change their resource use strategy under nitrogen addition, but their growth was inhibited (Figure 1). In our study, tall forbs have large roots (Figure 3a, higher root length and root area), which are physiologically relevant to root function, in particular to the uptake and conservation of water and nutrients [29]. These characteristics may make tall forbs insensitive to nitrogen addition. Nitrogen addition may reduce the growth of tall forbs by influencing environmental factors. We found a negative relationship between soil C/N and total biomass in tall forbs (Figure 4). Previous research has suggested that N addition affected plant growth by altering the availability of soil nutrients [13,16]. In our study, N addition increased ammonium (NH_4_^+^-N), especially for graminoids and tall forbs, which are consistent with N addition experiments [30]. Usually, plants freely absorb nitrogen in the form of ammonium and nitrate, but elevated ammonium concentrations can cause toxicity and inhibit plant growth [31,32]. Thus, there were negative correlations between ammonium (NH_4_^+^-N) and total biomass (Figure 4). However, these negative correlations were not significant, which may be due to the short study period. Previous studies have shown that the effects of N addition would be more pronounced over time [33]. In our study, soil properties had a relatively minor impact on plant growth and aesthetic value, which might be because other soil properties that we have not paid attention to have a stronger influence. For example, some studies found that soil acidification significantly affected the growth of different plants [12,13,34]. Future research should consider the impact of N addition on the soil acidification of green roofs. Considering that these modules only tested one growing season, we likely underestimate the specific responses of plant functional groups to N addition. In addition, seasonal and interannual variability, especially changes in rainfall and temperature, affect the growth of different plant species [2,35], and future research should also focus on the interactions between seasonal (interannual) variability and global climate change.

Compared to soil properties, plant functional traits can better explain changes in plant growth performance (Figure 4 and Figure S2). Plants with a resource-acquisition strategy are expected to grow better in high-resource environments, while resource-conservative plants are considered more adept and grow better in resource-scarce environments [27]. Considering nutrient leaching and its impact on water quality [3,36], we did not add any additional nutrients, except for 15% peat. So, the substrate fertility used for green roofs is usually low, which would limit the growth of plants. Combined with the water deficit faced by this habitat in North China, the rooftop would be unfavorable for the growth of resource-acquisition plants [37,38], such as graminoids and tall forbs. In our study, conservative traits (i.e., PC1 traits) in roots and leaves promoted plant growth in most cases (Figure 4). In graminoids, conservative traits (i.e., PC2 traits) did not increase plant growth, while acquisitive traits (i.e., PC1 traits) in leaves promoted belowground biomass through the transfer of photosynthetic products. In tall forbs, acquisitive traits (i.e., PC2 traits) in roots promoted aboveground biomass but inhibited belowground biomass (Figure 4a). These results indicated that the resource use strategies can be directly linked to plant growth [39] and that different plant functional groups exhibit variations in their adaptation to environments. Therefore, when trait data are available, plant candidates can be selected based on the resource use strategies represented by the traits [4].

The aesthetics of green roofs play a crucial role in their long-term acceptance by urban residents [3,6]. Throughout the entire experiment, all species survived, and most species from four plant functional groups were able to maintain a good relative appearance (i.e., RA near the value of 2). N addition decreased aesthetic value of sod-forming graminoids and tall forbs, which was similar to the change in total biomass. In this study, the high aesthetic value showed a combination of bright colors, compact structure, and vitality, which indicated that the plant is in a healthy state. Thus, plants with high aesthetic value had better growth performance (total biomass), except for succulents (Figure 4b–e). This may be because succulents have plenty of parenchyma in their stems, leaves, and roots for storing water and nutrients [23] and can sustain life and performance for a long time even under stress. We found a negative relationship between soil C/N and relative appearance in succulents, which indicated that high soil N improved plant vigor and aesthetic value [18]. We also demonstrated that functional traits could explain the changes in aesthetic value, except for tall forbs (Figure 4 and Figure S2). Specifically, long-lived leaves and roots promoted relative appearance of creeping forbs, while large leaves with high photosynthesis and large roots promoted relative appearance of sod-forming graminoids (Figure S2). For succulents, small and thick leaves, and roots with higher water acquisition promoted relative appearance (Figure S2).

In fact, the reduction in biomass under high N addition would limit the insulation capacity, retention capacity of active nitrogen, and carbon-storage capacity, which are considered ecological benefits of implementing green roofs [6,19,36]. Meanwhile, the aesthetic value loss caused by high nitrogen addition also reduced the attractiveness of these plant functional groups to urban residents [3]. We also found that nitrate nitrogen is beneficial for the creeping growth of the upper part of creeping forbs. Creeping forbs usually preferentially absorb nitrate nitrogen and expand their absorption of shallow nitrate nitrogen through creeping stems, promoting their photosynthetic capacity [16,17]. Thus, creeping forbs showed an increasing trend in biomass (Figure 1a,c). In such a scenario, creeping forbs and succulents, which can maintain stable growth and absorb nitrogen and reduce leaching of nitrogen from green roofs under N addition, are potential candidates for green roof implementation.

## 4. Materials and Methods

The study was carried out on a four-story building about 10 m high at Nankai University, Tianjin, China (38°59′16.08″N, 117°19′52.32″E). The study area has a temperate monsoon climate characterized by hot summers and cold winters. In this region, the annual average temperature is 13.8 °C, and the annual average precipitation is 704.5 Mm, respectively [8]. The average annual sunshine duration is 2470.9 h, with the higher values occurring from June to October (averaging approximately 222.2 h) [40]. Based on the data from 1980 to 2015, the total N deposition was approximately 35 kgN ha^−1^ yr^−1^ [11].

### 4.1. Plant Material

Eleven species were selected according to their life cycle type (perennial species), natural habitat (survive in shallow and/or barren soil), and aesthetic interest. These species belonged to four functional groups based on their similar morphologies and growth patterns (Table 1), including creeping forbs (*Duchesnea indica*, *Potentilla reptans*), sod-forming graminoids (*Buchloe dactyloides*, *Carex duriuscula*), succulents (*Sedum lineare*, *Sedum aizoon*, *Hylotelephium erythrostictum*), and tall forbs (*Iris tectorum*, *Hemerocallis fulva*, *Coreopsis drummondii*, *Physostegia virginiana*).

### 4.2. Experimental Design

In our experiment, we used customized green roof modules (30 cm × 30 cm × 25 cm, Figure S1), which were composed of a growing medium layer, a filter membrane (fabric filter about 2 mm thickness), a drainage system (30 mm height), and a root barrier layer (HDPE about 1.14 mm thickness) [8]. The substrate was composed of 40% pumice, 35% sand, 15% peat, and 10% vermiculite by volume. The initial nutrient contents of the substrate are relatively low (65.27 ± 3.52 mg g^−1^ for organic carbon, 4.58 ± 0.28 mg kg^−1^ for dissolved inorganic nitrogen, and 1.47 ± 0.27 mg kg^−1^ for available phosphorus in the substrate). A depth of 15 cm in each module was chosen for each module. All modules had the same growing substrate; only the planted vegetation differed between modules. Modules and substrate details are described in Supplementary Materials S1.

Two N addition treatments (3.5 gN m^−2^ yr^−1^ for low N addition, 10.5 gN m^−2^ yr^−1^ for high N addition) and a control treatment (0 gN m^−2^ yr^−1^) were simulated, and there were 33 modules per treatment (total 99 modules). Within each N addition group, one individual from each of the eleven species was randomly selected and planted into a module, with three replicates per species. The N addition treatment was conducted from 18 July to 21 October 2021. In the middle of each month, NH_4_NO_3_ was dissolved in 300 mL of water and added to the low-N and high-N addition treatments, while the control group received an equivalent volume of water. During the experiment, when there was no rainfall within a week, we carried out supplemental irrigation on the roof. Supplemental irrigation was provided equally to each module during the experiment.

### 4.3. Growth Measurements

At the end of the growing season, we harvested the aboveground and belowground parts of plant species for biomass calculation. Subsequently, the plants were oven-dried at 75 °C for 48 h until a constant weight was achieved. The total biomass (TB) was the sum of the aboveground (AB) and belowground biomass (BB). For plant cover, we took vertical pictures of the plants within each module using digital equipment. After geometric distortion correction and background interference removal, the proportion of plant pixel points to the total pixel points was calculated as an estimate of plant cover [4].

### 4.4. Aesthetic Evaluation

To evaluate the aesthetic value of plant species, some parameters considered in previous studies were adopted, such as leaf and stem color, number of leaves, stress states, plant survival, and vitality. Finally, three aesthetic parameters were selected, including plant and leaf color, plant shape, and plant vitality [3,5]. Before conducting the biomass collection, each parameter was observed and scored using a rating scale of one to three in each module. To minimize subjectivity, each scale value for the three parameters was linked to a distinct aspect of the plant. For plant color, a score of 3 represents optimal color richness, a score of 2 represents acceptable color richness, and a score of 1 represents aging or brown leaves. For plant shape, a score of 3 represents a compact shape, a score of 2 represents a partially open shape, and a score of 1 represents an amorphous plant. For plant vitality, a score of 3 indicates leaf swelling, a score of 2 indicates minimal leaf dehydration, and a score of 1 indicates that a few leaves are dehydrated. We calculated the relative appearance (RA) score as aesthetic value, that is, the average of the above three parameters.

### 4.5. Functional Trait Measurement

Considering the absorption, utilization and storage of light, and nutrients and water under N enrichment, we selected diverse functional traits that are closely related to these processes (Table S1), including leaf area (LA), leaf length (LL), leaf width (LW), leaf thickness (LT), leaf volume (LV), specific leaf area (SLA), leaf dry matter content (LDMC), leaf nitrogen content (LNC), leaf carbon content (LCC), leaf C/N ratio (LCN), plant height (H), root length (RL), root area (RA), root tissue density (RTD), specific root length (SRL), specific root area (SRA), root N content (RNC), root carbon content (RCC), and root C/N ratio (RCN). For example, SRL and SRA are closely correlated with assimilate utilization, nutrient uptake, and space niche in the canopy, while RTD is closely correlated with water transport and resource storage (Table S1).

We measured these functional traits of each species from 21 to 28 October 2021 under each N addition treatment. For each trait, three individuals from different modules were selected, then one leaf or root per individual was analyzed using the standard methods of Pérez-Harguindeguy et al. [41]. Fully expanded, young and healthy, undamaged leaves per species and fresh roots were selected for trait measurement. Briefly, fresh leaf and root mass were measured, and scanned to calculate LL, LW, and LA using ImageJ software (version 1.51j8) and to calculate RL, RA, and root volume using the WinRhizo root analysis system (version 2013e, Regent Instruments Inc., Canada). Then, leaf and root samples were oven-dried at 75 °C for 48 h to estimate the dry mass. SLA and SRA are the ratios of LA or RA to the dry mass of a leaf or root. LDMC is the ratio of leaf dry mass to fresh mass, and RTD is the ratio of root dry mass to root volume. Dry leaves or roots were powdered for the measurement of LCC, RCC, LNC, and RNC using an elemental analyzer. LCN and RCN are the ratios of LCC/RCC to LNC/RNC. For detailed information on trait measurement, refer to Supplementary Materials S1.

### 4.6. Soil Properties Measurement

Soil physicochemical properties were assessed using established soil analysis methods [42]. Prior to harvest, substrate moisture (SM) and temperature (Tem) in each module were measured using a ProCheck (Decagon Devices, Inc., Pullman, WA, USA). After harvest, substrate from each module was collected and analyzed for organic carbon (SOC), available phosphorus (AP), ammonium-N (NH_4_^+^-N), and nitrate-N (NO_3_^−^-N) content. SOC was measured using the potassium dichromate method, and AP was assayed using the Olsen method. NH_4_^+^-N and NO_3_^−^-N content was measured using the KCl extraction method. C/N ratio was calculated as the ratio of substrate C and N.

### 4.7. Statistical Analyses

The normality of the data was tested using the Kolmogorov–Smirnov test, and the homogeneity of the variances was determined using Levene’s test. A two-factor analysis of variance (ANOVA) using SPSS v20.0 (SPSS Inc., Chicago, IL, USA) was conducted to examine the effects of N enrichment and plant functional groups on the growth performance (AB, BB, and TB), plant cover, aesthetic value (RA), and soil properties (SM, Tem, SOC, AP, NO_3_^−^-N, NH_4_^+^-N, and CN). Then, the significance of differences among treatments was assessed using Tukey’s post hoc analysis.

In addition, principal component analyses (PCA) were conducted to characterize variations in plant traits across different plant functional groups. Then, regression analysis was used to assess the changes in plant traits (i.e., PC axes) with increased nitrogen addition. Correlation analysis and redundancy analysis (RDA) were performed to illustrate the relationship between growth performance, aesthetic value, and predictor variables (soil properties, plant functional traits) of different plant functional groups. The above analysis was conducted using ‘corrplot’ and ‘vegan’ packages in R4.1.2.

## 5. Conclusions

Our findings demonstrate that N addition had different effects on four plant functional groups, and decreased growth performance and aesthetic value of sod-forming graminoids and tall forbs. Nitrogen addition affected plant functional groups by altering plant resource utilization strategies. This study provides a valuable reference for plant selection in green roofs in temperate regions or other areas experiencing high nitrogen deposition, especially when selecting plant functional groups with conservative functional traits. It is worth noting that the robustness of these results needs to be tested on a longer time scale. Considering that monoculture of different plant functional groups was susceptible to environmental changes, combining different plant functional groups might achieve optimal growth and ecological benefits through plant species complementarity [8,43]. Further work should examine the suitability of different plant functional groups under different environmental changes to facilitate optimization of green roof performance in different regions, and analyze the changes in growth performance under the mixtures of different plant functional groups under environmental changes. In addition, given the rapid adoption of green roof systems in global cities, particularly with the increase in rooftop agriculture activities, evaluating the suitability of food species is also a future research focus under environmental changes [44].

## Figures and Tables

**Table 1:** The plant species information examined in this study includes the family name, natural habitat, and the functional group they belong to. Functional group: creeping forbs, dicotyledonous perennials with relatively broad leaves with prostrate growth; sod-forming graminoids, narrow-leaved monocotyledonous perennials with belowground rhizomes forming a dense sod; succulents, herbaceous perennials with succulent leaves and stems storing water; tall forbs, broad-leaved dicotyledonous perennials with erect growth.

Species	Family	Natural Habitat	Functional Group
*Duchesnea indica*	Rosaceae	Under the hillside forest	Creeping forbs
*Potentilla reptans*	Rosaceae	Hillside grassland and forest edge	Creeping forbs
*Carex duriuscula*	Cyperaceae	Semiarid grassland	Sod-forming graminoids
*Buchloe dactyloides*	Poaceae	Dry grasslands	Sod-forming graminoids
*Sedum lineare*	Crassulaceae	Rock crevices and hillside	Succulents
*Phedimus aizoon*	Crassulaceae	Hillside rocks and wasteland	Succulents
*Hylotelephium erythrostictum*	Crassulaceae	Hillside grassland, ditch edge, rock crevices	Succulents
*Hemerocallis fulva*	Asphodelaceae	Widespread habitat	Tall forbs
*Coreopsis basalis*	Asteraceae	Poor soils and sparse woods	Tall forbs
*Physostegia virginiana*	Lamiaceae	Sandy soil	Tall forbs
*Iris tectorum*	Iridaceae	Widespread habitat	Tall forbs

## Data Availability

The original contributions presented in this study are included in the Supplementary Materials. Further inquiries can be directed to the corresponding authors.

## Supplementary Materials

The following supporting information can be downloaded at: https://www.mdpi.com/article/10.3390/plants15010043/s1, Figure S1: Composition structure of modules in this study; Figure S2: Correlation analyses for individual functional trait and aboveground, belowground, total biomass, cover and esthetical value of creeping forbs, sod-forming graminoids, succulents and tall forbs; Table S1: List of 19 plant functional traits in this study. Refs. [41,45,46,47,48,49,50,51,52,53,54,55,56,57,58,59,60,61] have been cited in the supplementary materials.

## References

[1] Yang N., Guo Y., Zhang X., Hao G. (2023). Economy-society-ecology-humanistic values of green roofs and its contribution to sustainable development. South China Agr..

[2] Dunnett N., Nagase A., Hallam A. (2008). The dynamics of planted and colonising species on a green roof over six growing seasons 2001–2006: Influence of substrate depth. Urban Ecosyst..

[3] Azeñas V., Janner I., Medrano H., Gulías J. (2019). Evaluating the establishment performance of six native perennial mediterranean species for use in extensive green roofs under water-limiting conditions. Urban For. Urban Green..

[4] Hao G., Yang N., Chen X., Du Z., Li M., Chen L., Li H. (2024). Nitrogen enrichment decrease green roof multifunctionality. Urban For. Urban Green..

[5] Colleen B., Colin M.O. (2011). Sedum cools soil and can improve neighboring plant performance during water deficit on a green roof. Ecol. Eng..

[6] MacIvor J.S., Lundholm J. (2011). Performance evaluation of native plants suited to extensive green roof conditions in a maritime climate. Ecol. Eng..

[7] Nagase A., Dunnett N.P. (2012). Amount of water runoff from different vegetation types on extensive green roofs: Effects of plant species, diversity and plant structure. Landsc. Urban Plan..

[8] Hao G., Chen X., Du Z., Yang N., Li M., Gao Y., Liang J., Chen L., Li H. (2025). Phylogenetic and functional diversity consistently increase engineered ecosystem functioning under nitrogen enrichment: The example of green roofs. Ecol. Eng..

[9] Getter K., Rowe D. (2006). The role of extensive green roofs in sustainable development. HortScience.

[10] Eksi M., Rowe D.B. (2019). Effect of substrate depth and type on plant growth for extensive green roofs in a Mediterranean climate. J. Green Build..

[11] Yu G., Jia Y., He N., Zhu J., Chen Z., Wang Q., Piao S., Liu X., He H., Guo X. (2019). Stabilization of atmospheric nitrogen deposition in China over the past decade. Nat. Geosci..

[12] Tian Q., Liu N., Bai W., Li L., Chen J., Reich P.B., Yu Q., Guo D., Smith M.D., Knapp A.K. (2016). A novel soil manganese mechanism drives plant species loss with increased nitrogen deposition in a temperate steppe. Ecology.

[13] You C., Wu F., Gan Y., Yang W., Hu Z., Xu Z., Tan B., Liu L., Ni X. (2017). Grass and forbs respond differently to nitrogen addition: A meta-analysis of global grassland ecosystems. Sci. Rep..

[14] Shen H., Dong S., Li S., Xiao J., Han Y., Yang M., Zhang J., Gao X., Xu Y., Li Y. (2019). Effects of simulated N deposition on photosynthesis and productivity of key plants from different functional groups of alpine meadow on Qinghai-Tibetan plateau. Environ. Pollut..

[15] Stevens C.J., Dise N.B., Gowing D.J.G., Mountford O. (2006). Loss of forb diversity in relation to nitrogen deposition in the UK: Regional trends and potential controls. Glob. Change Biol..

[16] Zhang R., Shen H., Dong S., Li S., Xiao J., Zhi Y., Zhang J., Zuo H., Wu S., Mu Z. (2022). Effects of 5-year nitrogen addition on species composition and diversity of an alpine steppe plant community on Qinghai-Tibetan Plateau. Plants.

[17] Xu X., Liu H., Song Z., Wang W., Hu G., Qi Z. (2015). Response of aboveground biomass and diversity to nitrogen addition along a degradation gradient in the Inner Mongolian steppe, China. Sci. Rep..

[18] Clark M.J., Zheng Y. (2014). Effect of fertilizer rate on plant growth and leachate nutrient content during production of Sedum-vegetated green roof modules. HortScience.

[19] Dusza Y., Barot S., Kraepiel Y., Lata J., Abbadie L., Raynaud X. (2017). Multifunctionality is affected by interactions between green roof plant species, substrate depth, and substrate type. Ecol. Evol..

[20] Guo B., Arndt S., Miller R., Lu N., Farrell C. (2021). Are succulence or trait combinations related to plant survival on hot and dry green roofs?. Urban For. Urban Green..

[21] Siebenkäs A., Schumacher J., Roscher C. (2015). Phenotypic plasticity to light and nutrient availability alters functional trait ranking across eight perennial grassland species. AoB Plants.

[22] Pang Y., Tian J., Liu L., Han L., Wang D. (2021). Coupling of different plant functional group, soil, and litter nutrients in a natural secondary mixed forest in the Qinling Mountains, China. Environ. Sci. Pollut. Res..

[23] Grace O.M. (2019). Succulent plant diversity as natural capital. Plants People Planet.

[24] Yin H., Li M., Li D., Ali Khan S., Hepworth S.R., Wang S. (2019). Transcriptome analysis reveals regulatory framework for salt and osmotic tolerance in a succulent xerophyte. BMC Plant Biol..

[25] Cao F., Liu R., Huang G., Wu H., Zhao C. (2021). Effect of short-term nitrogen addition on productivity and plant diversity of subalpine grassland in Qilian Mountains. Acta Ecol. Sin..

[26] Lundholm J., Tran S., Gebert L. (2015). Plant functional traits predict green roof ecosystem services. Environ. Sci. Technol..

[27] Reich P.B. (2014). The world-wide ‘fast-slow’ plant economics spectrum: A traits manifesto. J. Ecol..

[28] Fort F., Cruz P., Jouany C. (2014). Hierarchy of root functional trait values and plasticity drive early-stage competition for water and phosphorus among grasses. Funct. Ecol..

[29] Craine J.M., Froehle J., Tilman D.G., Wedin D.A., Chapin F.S. (2001). The relationships among root and leaf traits of 76 grassland species and relative abundance along fertility and disturbance gradients. Oikos.

[30] Stuanes A.O., Kjonaas O.J. (1998). Soil solution chemistry during four years of NH4NO3 addition to a forested catchment at Grdsjn, Sweden. For. Ecol. Manag..

[31] Hachiya T., Sakakibara H. (2017). Interactions between nitrate and ammonium in their uptake, allocation, assimilation, and signaling in plants. J. Exp. Bot..

[32] Li G., Wang Z., Zhang L., Kronzucker H.J., Chen G., Wang Y., Shi W., Li Y. (2025). The role of the nitrate transporter NRT1.1 in plant iron homeostasis and toxicity on ammonium. Environ. Exp. Bot..

[33] Zhang X., Hasi M., Li A., Tan Y., Daryanto S., Wang L., Zhang X., Chen S., Huang J. (2021). Nitrogen addition amplified water effects on species composition shift and productivity increase. J. Plant Ecol..

[34] Tian Y., Bai X., Zhang X., Wei Z., Chen R., Niu X. (2017). Physiological response of four wild Poa to soil pH. Pratacultural Sci..

[35] Yee E.G., Callahan H.S., Griffin K.L., Palmer M.I., Lee S. (2022). Seasonal patterns of native plant cover and leaf trait variation on New York City green roofs. Urban Ecosyst..

[36] Johnson C., Schweinhart S., Buffam I. (2016). Plant species richness enhances nitrogen retention in green roof plots. Ecol. Appl..

[37] Yang S., Luo J., Tan N., Li X., Luo H., Zeng F., Liu X., Wu T. (2024). Effects of drought stress on growth, nutrient content, and stoichiometry of four native tree species in South China. Chinese J. Appl. Environ. Biol..

[38] Zhang Q., Hao G., Li M., Li L., Kang B., Yang N., Li H. (2022). Transformation of plant to resource acquisition under high nitrogen addition will reduce green roof ecosystem functioning. Front. Plant Sci..

[39] Lundholm J., Heim A., Tran S., Smith T. (2014). Leaf and life history traits predict plant growth in a green roof ecosystem. PLoS ONE.

[40] Guo J., Ren G. (2006). Variation characteristics of sunshine duration in Tianjin in recent 40 years and influential factors. Meteorol. Sci. Technol..

[41] Pérez-Harguindeguy N., Díaz S., Garnier E., Lavorel S., Poorter H., Jaureguiberry P., Bret-Harte M.S., Cornwell W.K., Craine J., Gurvich D.E. (2013). New handbook for standardised measurement of plant functional traits worldwide. Aust. J. Bot..

[42] Bao S.D. (2000). Soil and Agricultural Chemistry Analysis.

[43] Lundholm J.T., MacIvor J.S., MacDougall Z., Ranalli M.A. (2010). Plant species and functional group combinations affect green roof ecosystem functions. PLoS ONE.

[44] Leite F.R., Antunes M., Silva D. (2025). Sustainable construction based on green roofs designed to retain rainwater and provide food: An LCA compared to conventional roofs. Sustain. Prod. Consump..

[45] Anderson J.M., Ingram J.S.I. (1994). Tropical soil biology and fertility: A handbook of methods. Soil Sci..

[46] Bauer G.A., Bazzaz F.A., Minocha R., Long S., Magill A.H., Aber J.D., Berntson G.M. (2004). Effects of chronic N additions on tissue chemistry, photosynthetic capacity, and carbon sequestration potential of a red pine (*Pinus resinosa* Ait.) stand in the NE United States. Forest Ecol. Manag..

[47] Bryant J.P., Chapin F.S., Klein D.R. (1983). Carbon/nutrient balance of boreal plants in relation to vertebrate herbivory. Oikos.

[48] Chen Z.G., Batu N., Xu Z.Y., Hu Y.F. (2014). Measuring grassland vegetation cover using digital camera images. Acta Pratacult. Sin..

[49] Ding J., Kong D., Zhang Z., Cai Q., Xiao J., Liu Q., Yin H. (2020). Climate and soil nutrients differentially drive multidimensional fine root traits in ectomycorrhizal-dominated alpine coniferous forests. J. Ecol..

[50] Garnier E., Cortez J., Billès G., Navas M.L., Roumet C., Debussche M., Laurent G., Blanchard A., Aubry D., Bellmann A. (2004). Plant functional markers capture ecosystem properties during secondary succession. Ecology.

[51] Li Y., Niu S., Yu G. (2016). Aggravated phosphorus limitation on biomass production under increasing nitrogen loading: A meta-analysis. Global Change Biol..

[52] Lu R.K. (2000). Agriculture Chemical Analysis Methods of Soil.

[53] Maire V., Gross N., Börger L., Proulx R., Wirth C., Pontes L.D., Soussana J., Louault F. (2012). Habitat filtering and niche differentiation jointly explain species relative abundance within grassland communities along fertility and disturbance gradients. New Phytol..

[54] Nadelhoffer K.J., Emmett B.A., Gundersen P., Kjønaas O.J., Koopmans C.J., Schleppi P., Tietema A., Wright R. (1999). Nitrogen deposition makes a minor contribution to carbon sequestration in temperate forests. Nature.

[55] Ordoñez J.C., Van Bodegom P.M., Witte J.P.M., Wright I.J., Reich P.B., Aerts R. (2009). A global study of relationships between leaf traits, climate and soil measures of nutrient fertility. Global Ecol. Biogeogr..

[56] Rowe D.B., Getter K.L., Durhman A.K. (2012). Effect of green roof media depth on Crassulacean plant succession over seven years. Landscape Urban Plan..

[57] Wang H., Tang Y. (2016). Comparison of FIA and UV methods in determining soil nitrate nitrogen. J. Hebei Agr. Sci..

[58] Wang L., Wang J., Liu W.W., Gan Y., Wu Y. (2016). Biomass Allocation, Compensatory Growth and Internal C/N Balance of Lolium perenne in Response to Defoliation and Light Treatments. Pol. J. Ecol..

[59] Wilson J.B. (1988). A review of evidence on the control of shoot: Root ratio. Ann. Bot..

[60] Yang Y.H., Fang J.Y., Ji C.J., Han W.X. (2009). Above- and belowground biomass allocation in Tibetan grasslands. J. Veg. Sci..

[61] Zhan H., Yan S., Wang J., Ma C., Gong Z., Dong S., Zhang Q. (2015). Effect of returning rice straw into the field on soil phosphatase activity and available phosphorus content. Crop J..

