# Supplementary material for: Evaluating the Suitability of Four Plant Functional Groups in Green Roofs Under Nitrogen Deposition
Source: Plants (Basel). 2025 Dec 23;15(1):43. doi: 10.3390/plants15010043 (PMC12787474; doi:10.3390/plants15010043)
Supplement: Supplementary file 1 [file plants-15-00043-s001.zip › plants-3996252-supplementary.pdf]

Appendix A Supplementary data

Extra information on methods and results

belongs to the study:

Evaluating the suitability of four plant functional groups in green roofs under nitrogen deposition

Nan Yang, Hechen Li, Runze Wu, Yihan Wang, Meiyang Li, Lei Chen, Hongyuan Li, Guang Hao

S1 Methods

Setup of substrate

Ninety-nine modules (30 cm × 30 cm × 30 cm) were customized (supplied by Jinhu Landscape Engineering Co., Ltd., in Tianjin) on May 2021 (Fig. S1). A high-density polyethylene (HDPE) membrane root barrier layer of 1.14 mm thickness continuously covered the entire wood deck and was laid directly below the drainage panel (30 mm height). This dimpled drain board comes in a single roll with water capacity of 4.84 L m^{-2} and flow rate (core) 0.25 L s^{-1} m^{-1}, which was engineered to provide the balance of retention and air movement in the green roof modules. A fabric filter of 2 mm thickness with 65 L s^{-1} m^{-2} water flow capacity was laid over the drainage panel to keep the growing media in place and discourage intrusion by plant roots. The growing medium layer was filled with a depth of 150 mm mixed substrate. The mixed substrate employed was made up of 40% pumice, 35% sand, 15% peat, and 10% vermiculite by volume (all these materials from Xuebin Horticulture Ltd., in Tianjin).

Measurement of plant traits

(i.e. control, low N enrichment and high N enrichment) using standard protocols (Pérez-Harguindeguy et al., 2013), including leaf area (LA), leaf length (LL), leaf width (LW), leaf thickness (LT), leaf volume (LV), specific leaf area (SLA), leaf dry matter content (LDMC), leaf nitrogen content (LNC), leaf carbon content (LCC), leaf C/N ratio (LCN), plant height (H), root length (RL), root area (RA), root tissue density (RTD), specific root length (SRL), specific root area (SRA), root N content (RNC), root carbon content (RCC) and root C/N ratio (RCN).

Before harvesting the whole plant, we measured plant height (H) from three individuals in different plots. Height is the vertical natural distance from the upper boundary of plant photosynthetic tissue to ground level. Fully expanded, young and healthy, undamaged leaves per species (one leaf per individual) were selected. Leaves were collected in wet plastic bags and transported to the lab. Then, fresh leaf mass was measured, and each of the three leaves per species was scanned. Leaf length

(LL), width (LW), and area (LA) was calculated using ImageJ software (version 1.51j8; National Institutes of Health, Bethesda, MD, United States). After that, the leaves were oven-dried at 75°C for 48 h to estimate the dry leaf mass. Then, we calculated specific leaf area (SLA, the ratio of LA to the leaf dry mass) and leaf dry matter content (LDMC, the ratio of leaf dry mass to fresh mass). Leaf thickness (LT) was calculated as 1/(SLA×LDMC), and leaf volume (LV) was calculated as LA × LT. Finally, dry leaves were powdered for the measurement of leaf carbon content (LCC) and leaf nitrogen content (LNC) using an elemental analyzer (Vario MAX C/N-Macro Elemental Analyzer) and then calculated LCN (LCN = LCC / LNC).

All species roots were collected from plots as completely as possible. After cleaning the fresh roots, 99 root samples (11 species × 3 duplicates × 3 N enrichment treatments) for the morphological and architectural measurements were scanned at 400 dpi using a scanner (EPSON Perfection V700/V750) and analyzed (WinRhizo root analysis system, Canada) to calculate the total root length (RL), root area (RA), specific root length (SRL) and specific root area (SRA). The corresponding root samples were dried at 75°C for 48 hours to calculated root tissue density (RTD). SRL was calculated as the root length per unit dry weight, SRA was calculated as the root area per unit dry weight, and RTD was calculated from root dry weight divided by its volume (Comas and Eissenstat, 2009). In the similar way, dry roots were powdered for the measurement of root carbon content (RCC) and root nitrogen content (RNC) using an elemental analyzer and then calculated RCN (RCN = RCC / RNC). Finally, for each species, three individuals in different plots (one individual per plot) were selected, and one leaf or root per individual was selected for trait measurement under each N enrichment treatment.

S2 Tables and Figures

Table S1 List of 19 plant functional traits in this study

**Trait**	**Unit**	**Characteristic**	**Function**
Aboveground traits			
Specific leaf area (SLA)	cm^2/g	Morphology	Assimilate utilization, light interception, space niche in canopy (Wilson et al., 1999; Rowe et al., 2012)
Leaf area (LA)	cm^2	Morphology	Light interception (Wilson et al., 1999; Maire et al., 2012)
Leaf length (LL)	cm	Morphology	Light interception, avoidance (Maire et al., 2012)
Leaf width (LW)	cm	Morphology	Light interception, avoidance (Maire et al., 2012)
Leaf thickness (LT)	cm	Morphology	Water storage (Garnier et al., 2004; Rowe et al., 2012)
Leaf volume (LV)	cm^3	Morphology	Space niche in canopy, Water retention (Maire et al., 2012)
Leaf dry matter content (LDMC)	-	Morphology	Assimilate utilization, decomposability (Maire et al., 2012)
Leaf N content (LNC)	mol/L	Chemical composition	Photosynthetic capacity (Yang et al., 2009; Ordoñez et al., 2009)
Leaf C content LCC)	mol/L	Chemical composition	Assimilate utilization, leaf architecture (Bryant et al., 1983; Maire et al., 2012)
Leaf C/N ratio (LCN)	-	Chemical composition	Leaf growth resource limitation (Wilson, 1988; Wang et al., 2016)
Plant height (H)	cm	Morphology	Light interception, avoidance (Walburg et al., 1982; Yin et al., 2011)
Belowground traits			

Specific root length (SRL)	cm/g	Morphology	Assimilate utilization, nutrient uptake, space niche in canopy, forage efficiency (Li et al., 2016; Ding et al., 2020)
Specific root area (SRA)	cm^2/g	Morphology	Assimilate utilization, nutrient uptake, space niche in canopy (Li et al., 2016)
Root length (RL)	cm	Morphology	Space niche in soil, nutrient acquisition strategy (Maire et al., 2012)
Root area per soil volume (RA)	cm^2	Morphology	Space niche in soil, nutrient acquisition strategy (Maire et al., 2012)
Root N content (RNC)	mol/L	Chemical composition	Efficiency of nitrogen transport and utilization (Nadelhoffer et al., 1999; Bauer et al., 2004)
Root C content (RCC)	mol/L	Chemical composition	Efficiency of carbon transport and utilization, root architecture (Bryant et al., 1983; Maire et al., 2012)
Root C/N ratio (RCN)	-	Chemical composition	Root growth resource limitation, soil nutrient resource limitation (Wilson, 1988; Wang et al., 2016)
Root tissue density (RTD)	g/cm^3	Morphology	Assimilate utilization, water transport, resource storage (Maire et al., 2012)

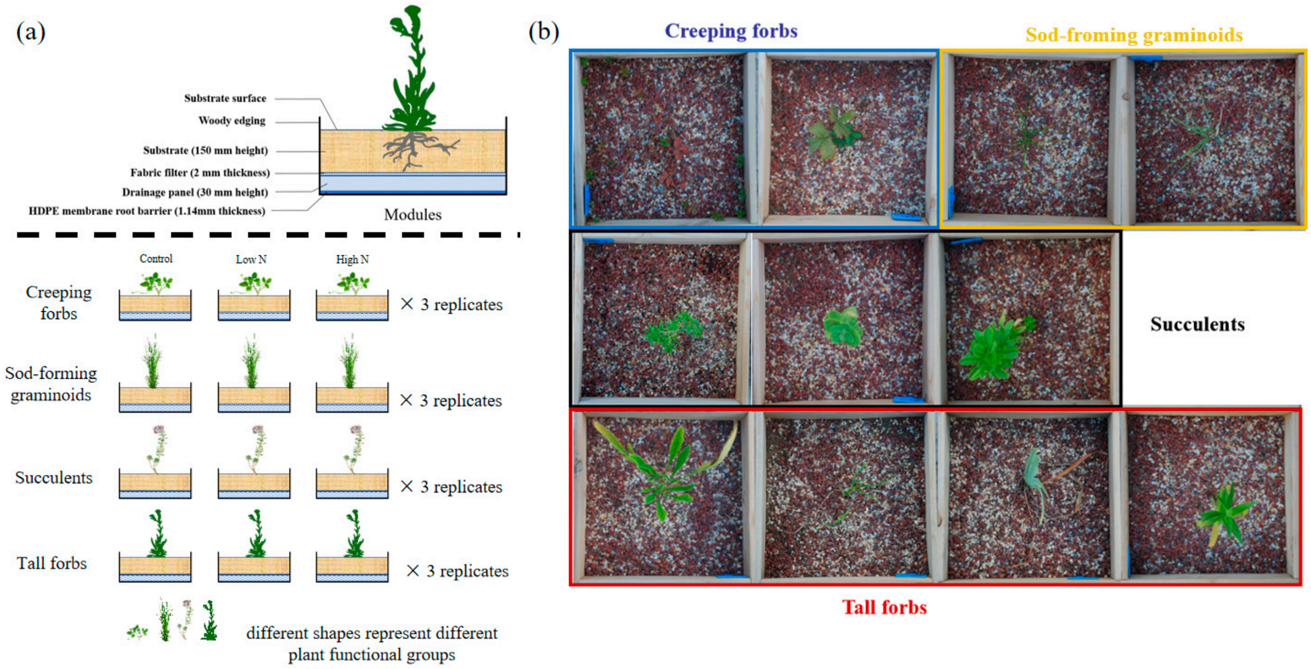

Fig. S1 Composition structure of modules and experimental design (a) and the photo of plant functional groups (b) in this study

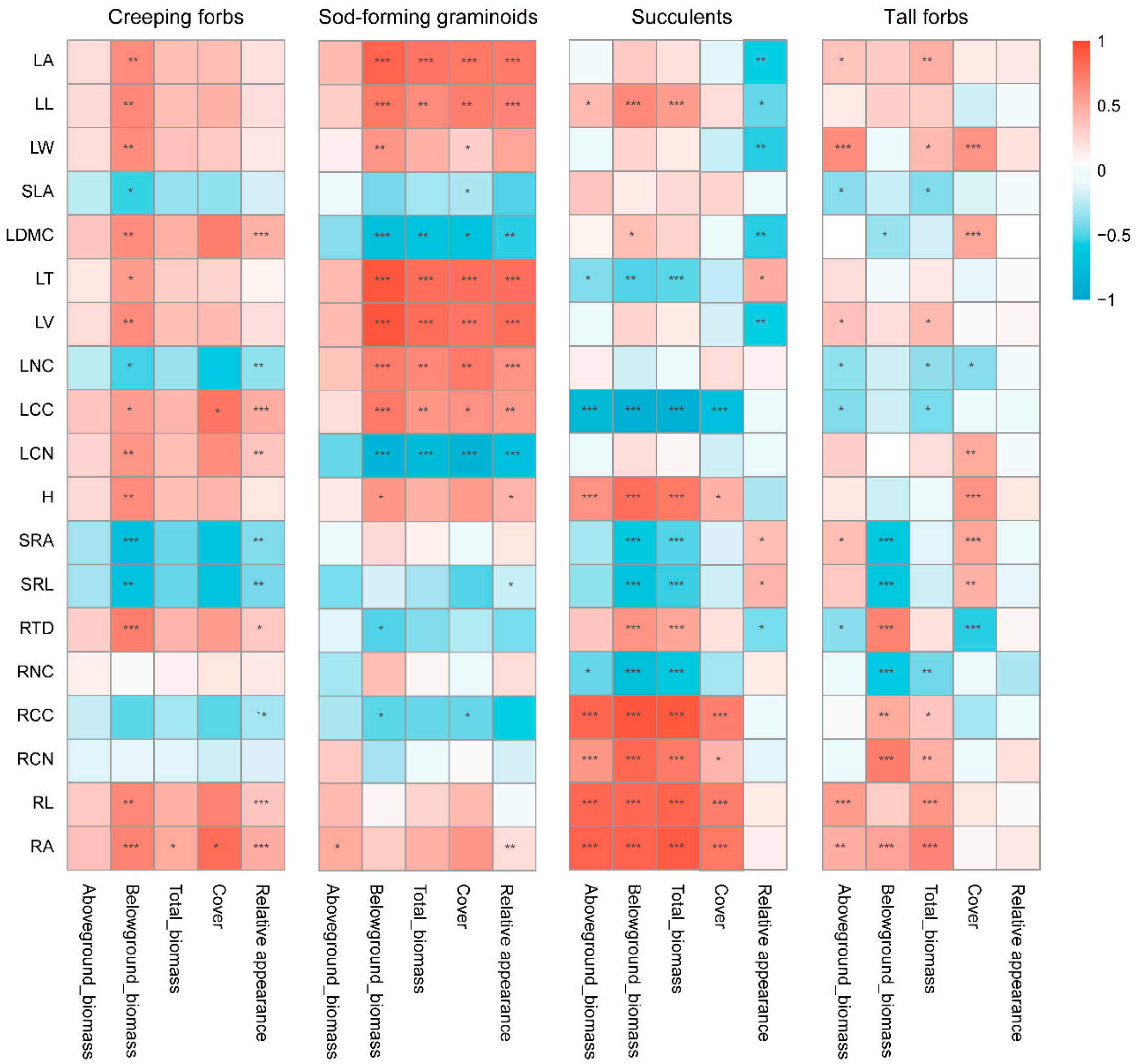

Fig. S2 Correlation analyses for individual functional trait and aboveground, belowground, total biomass, cover and esthetical value of creeping forbs, sod-forming graminoids, succulents and tall forbs. Red and blue colors represent positive and negative correlations, respectively. Significance level of correlations was represented by * (p < 0.05), ** (p < 0.01) and *** (p < 0.001).

References list

Anderson, J.M.; Ingram, J.S.I. Tropical Soil Biology and Fertility: A Handbook of Methods. *Soil Sci.* 1994, 157, 265.

Bauer, G.A.; Bazzaz, F.A.; Minocha, R.; Long, S.; Magill, A.H.; Aber, J.D.; Berntson, G.M. Effects of chronic N additions on tissue chemistry, photosynthetic capacity, and carbon sequestration potential of a red pine (*Pinus resinosa* Ait.) stand in the NE United States. *Forest Ecol. Manag.* 2004, 196, 173-186.

Bryant, J.P.; Chapin, F.S.; Klein, D.R. Carbon/nutrient balance of boreal plants in relation to vertebrate herbivory. *Oikos* 1983, 40, 357.

Chen, Z.G.; Batunacun, Xu Z.Y.; Hu Y.F. Measuring grassland vegetation cover using digital camera images. *Acta Prataculturae Sin.* 2014, 23, 20-27.

Ding, J.; Kong, D.; Zhang, Z.; Cai, Q.; Xiao, J.; Liu, Q.; Yin, H. Climate and soil nutrients differentially drive multidimensional fine root traits in ectomycorrhizal-dominated alpine coniferous forests. *J. Ecol.* 2020, 108, 2544-2556.

Garnier, E.; Cortez, J.; Billès, G.; Navas, M.L.; Roumet, C.; Debussche, M.; Laurent, G.; Blanchard, A.; Aubry, D.; Bellmann, A.; et al. Plant functional markers capture ecosystem properties during secondary succession. *Ecology* 2004, 85, 2630-2637.

Li, Y.; Niu, S.; Yu, G. Aggravated phosphorus limitation on biomass production under increasing nitrogen loading: a meta-analysis. *Global Change Biol.* 2016, 222, 934-943.

Lu, R.K. Agriculture chemical analysis methods of soil (in Chinese). Beijing, China: Agricultural Science and Technology Press, 2000.

Maire, V.; Gross, N.; Börger, L.; Proulx, R.; Wirth, C.; Pontes, L.D.; Soussana, J.; Louault, F. Habitat filtering and niche differentiation jointly explain species relative abundance within grassland communities along fertility and disturbance gradients. *New Phytol.* 2012, 1962, 497-509.

Nadelhoffer, K.J.; Emmett, B.A.; Gundersen, P.; Kjønaas, O. J.; Koopmans, C.J.; Schleppi, P.; Tietema, A.; Wright, R. Nitrogen deposition makes a minor contribution to carbon sequestration in temperate forests. *Nature* 1999, 398, 145-148.

Ordoñez, J.C.; Van Bodegom, P.M.; Witte, J.P.M.; Wright, I.J.; Reich, P.B.; Aerts, R. A global study of relationships between leaf traits, climate and soil measures of nutrient fertility. *Global Ecol. Biogeogr.* 2009,18, 137-149.

Pérez-Harguindeguy, N.; Díaz, S.; Garnier, E.; Lavorel, S.; Poorter, H.; Jaureguiberry, P.; Bret-Harte, M.S.; Cornwell, W.K.; Craine, J.; Gurvich, D.E.; et al. New handbook for standardised measurement of plant functional traits worldwide. *Aust. J. Bot.* 2013, 61, 167-234.

Rowe, D.B.; Getter, K.L.; Durhman, A.K. Effect of green roof media depth on Crassulacean plant succession over seven years. *Landscape Urban Plan.* 2012, 104, 310-319.

Wang, H.; Tang, Y. Comparison of FIA and UV methods in determining soil nitrate nitrogen. *J. Hebei Agr. Sci.* 2016, 20, 105-108.

Wang, L.; Wang, J.; Liu, W. W.; Gan, Y.; Wu, Y. Biomass Allocation, Compensatory Growth and Internal C/N Balance of Lolium perenne in Response to Defoliation and Light Treatments. *Pol. J. Ecol.* 2016, 64, 485-499.

Wilson, J.B. A review of evidence on the control of shoot: root ratio. *Ann. Bot.* 1988, 61, 433-449.

Yang, Y. H.; Fang, J. Y.; Ji, C. J.; Han, W.X. Above- and belowground biomass allocation in Tibetan grasslands. *J. Veg. Sci.* 2009, 20, 177–184.

Zhan, H.; Yan, S.; Wang, J.; Ma, C.; Gong, Z.; Dong, S.; Zhang, Q. Effect of returning rice straw into the field on soil phosphatase activity and available phosphorus content. *Crop J.* 2015, 000, 78-83.
